# Supplementary material for: Impact of Alzheimer’s disease risk factors and local neuromelanin content on the transcriptomic landscape of the human locus coeruleus
Source: bioRxiv. 2025 Oct 29:2025.10.29.685354. Preprint. [Version 1] doi: 10.1101/2025.10.29.685354 (PMC12636595; doi:10.1101/2025.10.29.685354)
Supplement: Supplement 2 [file media-2.pdf]

Supplemental Table Legends

Table S1. Donor demographics, demographic/analysis groups, and RNA-seq metrics from the filtered Visium data. A) Demographic variables and, where available, tau and Aß pathology ratings (*Methods*) for each donor. Ancestry is assigned based on predominant (≥50%) global genomic ancestry estimates (*Methods*). **B)** *N* and demographics for the donors included in DE and other downstream analyses (up to 30). Subgroupings are provided by sex, predominant ancestry, and *APOE* haplotype. Only those donors considered in DE and other downstream analyses (up to 30) are considered. **C)** Demographic and other variables frequently considered in RNA-seq analyses by Visium capture area. RNA integrity number (RIN) are listed for cortical tissue from the same donors (although dissection and freezing of brainstem is performed separately from cortex during tissue banking). Also provided are means and medians of spot-level RNA-seq metrics per capture area after removing low-content and local outlier spots.

Table S2. **Spatially variable genes (SVGs) and high-binomial deviance gene (HDGs) analyses; spatial registration[88–90] with human and mouse LC snRNA-seq data.** SVG sheets include a summary sheet of analysis, listing gene ranks for nominally significant genes in each sample, and separately including *nnSVG*[77] output for all genes analyzed in each sample. A third sheet provides full HDG analysis[76] results used to identify HDGs. The first three sheets contain a column to indicate genes used for clustering. Spatial registration results for the 9 reported domains with human[91] LC and mouse[87] RNA-seq datasets, calculated over a range of 10 to >1000 top-ranked marker genes for each reference cluster/domain.

Table S3. **Clustering QC metrics for the assigned domain labels; domain proportions by donor and tissue section.** The first sheet contains the spot identifier, assigned label, 3 columns of results from cluster purity analysis, a column for silhouette width, and a column for root mean-square deviation (one value per domain). The remaining sheets detail the proportion of spots assigned each domain by brain donor or by tissue section.

Table S4. **Domain marker gene analysis and domain comparisons among *ACHE-SERT*, Oligo, or Astro domains.** The first sheet provides the full results of one-vs-all marker analyses for the 9 reported domains. The remaining sheets include analogous results when only comparing among Astro domains, among Oligo domains, or among *ACHE-SERT* domains.

Table S5. **snRNA-seq marker analysis of *ACHE-SERT* domain-enriched inhibitory subcluster 4.** Marker analysis table for the inhibitory cluster from[91] predicted to be a large source of expression in *ACHE-SERT* domains by *RCTD*[92]. Also see **Fig. S10**.

Table S6. **nnSVG results from analysis restricted to the LC domain**. A summary sheet of the analysis, lists ranks for nominally significant genes in the LC domain of each sample, and separately including *nnSVG*[77] output for all genes analyzed in each sample. Note that some LC-containing samples with insufficient spots to determine spatial variation were not analyzed.

Table S7. **DE and GSEA analyses comparing LC^{NM+} and LC^{NM-}.** Sheet with DE results, where positive logFC signifies greater expression in LC^{NM+} relative to LC^{NM-}. A second sheet is concatenated results from two GSEA analyses (one examining MSigDB gene sets, one examining sets of TF-regulated genes). TF-target results were subsetted to those where the TF was at least marginally expressed in LC (see *Methods*). Additional information is included in the GSEA sheet to aid in table interpretation.

Table S8. ***SCORPION* GRN inferred LC^{NM+}/LC^{NM-}-specific gene-regulatory relationships and TFs biased toward LC^{NM+}/LC^{NM-}-specific activity.** A table of the ~1,669 GRN edges (TF-gene pairs) deemed LC^{NM+}/LC^{NM-}-specific (see *Methods*). A second sheet contains the 1,669 aforementioned edges plus all edges involving a TF with LC^{NM+}/LC^{NM-}-specific activity. The third sheet lists these TFs, which are those with ≥80% of edges specific to either subdomain being LC^{NM+} or LC^{NM-}-specific; that is, *# subdomain-specific edges* / *(# of* LC^{NM+} *specific edges + # of* LC^{NM-} *specific edges)*>0.8.

Table S9. **Genes unique to one-few LC NMF factors and Enrichr analysis of NMF factor-specific genes**. Genes identified as uniquely upweighted in 1-3 NMF factors are listed, with a row for each factor-gene pair. Enrichr results with an adjusted *p*-value<0.05 are provided alongside details to aid in table interpretation. The background gene list used for enrichment testing in this analysis is also included.

Table S10. **DE results from 5 domains compared between sexes, E4 and E2 controlling for ancestry, ancestry-stratified E4-E2 comparisons, and between predominant ancestries.** Each sheet's title indicates the comparison, with the group in which a positive logFC signifies upregulation listed first (e.g., 'male-female'). A legend sheet is also included.

Table S11. **MSigDB and TF-target GSEA analyses on sex-DE and haplotype-DE.** MSigDB GSEA results significant at an FDR<0.05 are included for each DE analysis. For TF-target analyses, results were subsetted to those where the corresponding TF of interest was expressed in the analyzed domain (see *Methods*). Sheets include a top row with column definitions and sign conventions to facilitate interpretation.

Table S12. **Ancestry-haplotype DE enrichments in MSigDB gene sets.** The first sheet lists those GSEA results that were significant (FDR<0.05) from E4-E2 analysis in a domain for only one ancestry. The remaining sheets provide all FDR<0.05 GSEA results from ancestry-specific haplotype-DE (AA E4-E2 and EA E4-E2) and single-ancestry haplotype-DE (E4 AA-EA and E2 AA-EA).

Table S13. **Results from linear mixed models of spot-level NM intensity or spot NM pixel proportion against spot-level gene expression.** Sheets provide results of the reported NM intensity modeling as well as modeling of NM pixel proportion. For each gene, rows are included with the coefficient and raw *p*-value for each fixed effect (sex, E4/E2 carrier, proportion African genomic ancestry, age, and gene expression) and for the log ratio test statistic comparing models of the NM metric with and without gene expression. For gene coefficients, the FDR- and Bonferroni-corrected p values are also listed. Sheets include additional details to aid in interpretation.

Table S14. ***Enrichr* analyses of NM intensity- or proportion-associated genes.** Top results from 6 *Enrichr* analyses per NM metric modeled: two significance cutoffs for NM association (FDR<0.05, Bonferroni *p*<0.05) and three bins by sign of association (positive, negative, all). *Enrichr* reports the rank of each test within a library of gene sets[105,175]; up to 20 significant enrichments are listed for each *Enrichr* library and input set of NM-associated genes. A sheet with the background list of genes for these analyses is also included.

Supplemental Data Legends

Supplemental Data S1. **Spotplots of domain clusters across samples.** Annotations are shown for each of 43 capture areas (generally 2 samples/tissue sections). Only spots retained after QC were assigned a domain; no annotation is plotted for spots removed by QC, revealing the H&E tissue beneath. Individual samples and annotations can be interactively viewed in uniform anatomic orientation using the provided resources in *Data Access and Visualization*.

Supplemental Data S2. **Spot deconvolution results using RCTD and previously described adult human LC-NE snRNA-seq**. An *.RDS* object is provided, with a named list object at the top level containing four different tabular results, where each row is a spot, each column is an snRNA-seq label, and each cell is an *RCTD*[92] weight, giving the estimated proportion of spot expression attributable to an snRNA-seq cell type. The list names indicate the *RCTD* mode used ("Multi"--deconvolution assuming a spot contains ≤4 cell types from the reference data, or "Full"--deconvolution with no limit on the number of cell types) and snRNA-seq cell annotation used ("label": 30 unnamed granular clusters; "label_merged": 10 named, broad cell types grouped from these 30 clusters). For readability, the granular 'labels' were concatenated with the cell type they were grouped with in 'label_merged' (e.g., the granular label 4 was grouped into 'inhibitory' under label_merged, and thus is presented here as "inhibitory_4").

Supplemental Data S3. **Complete *SCORPION* GRN for LC^{NM+} and LC^{NM-} with edge weight comparisons.** Unabridged version of the GRN edge comparisons in **Table S8**. Columns *nmp* and *nmn* provide the *Z*-normalized edge weights from LC^{NM+} and LC^{NM-} GRNs, respectively. Column *diff* is the edge weight difference, and nmp_spec/nmn_spec indicate edges determined to be specific to LC^{NM+} or LC^{NM-} (see *Methods*), respectively.

Supplemental Data S4. **Raw NMF matrices *w* and *h* resulting from decomposition of the LC domain into 160 factors.** An *.RDS* object is provided containing a list with two slots, "*w*" and "*h*", corresponding to gene weight and factor loading matrices. Factor, gene, and spot identifiers are included as row/column names.

Supplemental Data S5. **Complete *Enrichr*[105] results from analysis of genes associated with NM intensity or NM pixel proportion.** An .RDS list object with named slots containing the concatenated results from NM intensity or NM pixel proportion tests (6 tests per NM metric: FDR-significant or Bonferroni-significant genes and subsets thereof in each direction of association). The same tables filtered to top enrichments and with a key with more information for interpretation are in **Table S14**.
